# Experimental Benefits of Sex Hormones on Vascular Function and the Outcome of Hormone Therapy in Cardiovascular Disease

**DOI:** 10.2174/157340308786349462

**Published:** 2008-11

**Authors:** Reagan L Ross, Michelle R Serock, Raouf A Khalil

**Affiliations:** Division of Vascular Surgery, Brigham and Women’s Hospital, and Harvard Medical School, Boston, Massachusetts 02115, USA

**Keywords:** Estrogen, progesterone, testosterone, hypertension, coronary artery disease, endothelium, nitric oxide, vascular smooth muscle, calcium.

## Abstract

Cardiovascular disease (CVD) is more common in men and postmenopausal women than premenopausal women, suggesting vascular benefits of female sex hormones. Experimental data have shown beneficial vascular effects of estrogen including stimulation of endothelium-dependent nitric oxide, prostacyclin and hyperpolarizing factor-mediated vascular relaxation. However, the experimental evidence did not translate into vascular benefits of hormone replacement therapy (HRT) in postmenopausal women, and HERS, HERS-II and WHI clinical trials demonstrated adverse cardiovascular events with HRT. The lack of vascular benefits of HRT could be related to the hormone used, the vascular estrogen receptor (ER), and the subject’s age and preexisting cardiovascular condition. Natural and phytoestrogens in small doses may be more beneficial than synthetic estrogen. Specific estrogen receptor modulators (SERMs) could maximize the vascular benefits, with little side effects on breast cancer. Transdermal estrogens avoid the first-pass liver metabolism associated with the oral route. Postmenopausal decrease and genetic polymorphism in vascular ER and post-receptor signaling mechanisms could also modify the effects of HRT. Variants of cytosolic/nuclear ER mediate transcriptional genomic effects that stimulate endothelial cell growth, but inhibit vascular smooth muscle (VSM) proliferation. Also, plasma membrane ERs trigger not only non-genomic stimulation of endothelium-dependent vascular relaxation, but also inhibition of [Ca^2+^]i, protein kinase C and Rho kinase-dependent VSM contraction. HRT could also be more effective in the perimenopausal period than in older postmenopausal women, and may prevent the development, while worsening preexisting CVD. Lastly, progesterone may modify the vascular effects of estrogen, and modulators of estrogen/testosterone ratio could provide alternative HRT combinations. Thus, the type, dose, route of administration and the timing/duration of HRT should be customized depending on the subject’s age and preexisting cardiovascular condition, and thereby make it possible to translate the beneficial vascular effects of sex hormones to the outcome of HRT in postmenopausal CVD.

## INTRODUCTION

 The incidence of CVD is greater among men 35-50 years of age compared to women of similar age, and in postmenopausal compared to premenopausal women [1,2]. Experimental studies have identified ER in the cytosol and nucleus of the endothelium, VSM and adventitial cells of the vascular wall. Estrogen may activate the cytosolic/nuclear ERs, stimulate genomic effects, and induce changes in vascular cell growth and vascular tissue remodeling. Estrogen may also activate plasma membrane ERs and induce non-genomic effects on the endothelium and VSM [1,3,4]. Studies in animal models of hypertension have shown decreased blood pressure in ovariectomized (OVX) female rats treated with ER agonists [5]. Also, initial clinical observational studies have shown reduced incidence of CVD in postmenopausal women receiving HRT [6,7]. However, data from the Heart and Estrogen/Progestin Replacement Study (HERS), HERS-II, and the Women’s Health Initiative (WHI) clinical trials did not support beneficial effects of HRT in postmenopausal women [8-11] (Table **1**). The HERS trial enrolled postmenopausal women with established coronary artery disease and failed to demonstrate the benefits of estrogen on secondary cardiovascular prevention. Instead, there was an increase in cardiac events within the HRT group in the first year of the study [9,10]. The WHI study evaluated the effects of hormones on primary cardiovascular prevention in healthy postmenopausal women, and was terminated early due to a small but significant increase in cardiovascular events and adverse outcomes in the HRT group [11]. The Estrogen Replacement in Atherosclerosis (ERA) clinical trial has also shown no significant effect of HRT on coronary atherosclerosis as assessed by angiography [12]. These findings have directed the avoidance of HRT in postmenopausal women, at least in the context of CVD prevention. On the other hand, the disappointing outcome of HRT in clinical trials has also prompted more careful re-examination of the cardiovascular effects of sex hormones. 

The purpose of this review is to highlight the beneficial vascular effects of estrogen observed in experimental studies, and why translation of these effects into vascular benefits did not materialize in the HRT clinical trials. The lack of vascular benefits of HRT in postmenopausal women could be related to the hormone used, the vascular hormone receptors, and the subjects studied. In this review, factors related to the hormone type, dose, and route of administration will be described. Potential postmenopausal changes in vascular ER and post-receptor signaling mechanisms of vascular relaxation/contraction will also be discussed. Factors related to the timing/duration of HRT, the subject’s age and preexisting cardiovascular condition will be analyzed. The potential vascular effects of progesterone and testosterone will then be discussed. The review will finalize with a perspective on potential areas of research that could enhance the vascular benefits of HRT in menopausal women.

## TYPES OF HRT

The primary source of estrogen in normally cycling adult women is the ovarian follicle, which secretes 70 to 500 µg estradiol daily depending on the phase of the menstrual cycle. In adult women, the plasma levels of estrogen show significant fluctuations during the menstrual cycle from 210 pmol/L in the early follicular and 720 pmol/L in the late follicular phase to 490 pmol/L in the late luteal phase. During menopause the ovarian production of estradiol is significantly reduced and most endogenous estrogen is produced by conversion of androstenedione, secreted by the adrenal cortex, to estrone by peripheral tissues. In postmenopausal women, the plasma estrogen level is reduced to <100 pmol/L, making estrogen the primary and most logical component of HRT [13].

The type of estrogen used could affect the outcome of HRT in postmenopausal women. Natural estrogens have the same structure as the endogenous animal estradiol made by the ovaries, and are easily metabolized and excreted. On the other hand, semi-synthetic estradiol (estrace) and synthetic estrogens are the most widely prescribed. They are similar in structure to natural estrogens and have strong vascular effects, but that comes with unwanted adverse effects [14-16]. Examples of synthetic estrogens are diethylstilbestrol, ethinyl estradiol (Levlen), and estradiol benzoate (Celerin), cypionate and valerate. Mestranol is a prodrug that is quickly demethylated in the body to ethinyl estradiol.

Conjugated equine estrogen (CEE) is a synthetic estrogen and the most commonly prescribed estrogen supplements in the United States. CEE is synthesized from the urine of pregnant mares and is available in formulas like Premarin, Prempro, Premphase and Prempac. CEE has been used in most HRT clinical trials, and has been shown to inhibit aortic connective tissue remodeling after plasma lipid lowering in female monkeys [17]. However, CEE has also been linked to increased cancer rates

Most of what we learn about estrogen is in reference to the two principle estrogens produced in woman’s body, estradiol and estrone, and the horse-derived CEE. Estriol is one of the three natural estrogens produced in high levels during pregnancy as it is made by the placenta. Although estriol has been widely used in Europe for over fifty years, it has not received equal attention in the U.S. particularly with regards to its vascular effects. Estriol is the weakest of natural estrogens and has been used as an alternative to the more potent estradiol and estrone for managing menopausal symptoms [18]. Because estriol is less potent than estradiol and estrone it has little side-effects when used in low doses, and does not exhibit strong estrogenic effects such as endometrial and breast tissue growth. In a study examining clinically used estrogens, estriol, estrone and estrone sulfate did not cause as much inhibition of human aortic smooth muscle cell growth, proliferation and migration and reduction of mitogen-activated protein kinase activity as that induced by E2, estradiol valerate, estradiol cypionate, or estradiol benzoate [19]. 

“Hormone Bioidenticals” are promoted for managing postmenopausal symptoms such as hot flushes and mood swings; however, their vascular effects need to be evaluated. Bioidentical estriol is available as customized prescriptions. Using DNA engineering, sterol analogs found in plants such as wild yam are subjected to microbial fermentation and the resulting hormone is bioidentical in chemical structure to the natural estriol produced in women during pregnancy.

Phytoestrogens are chemicals found in plants that bind to ER and may act like natural estrogens. Phytoestrogens are thousand-fold weaker than estrogen, but circulate in the blood at levels thousand-fold higher than natural estrogens do [20,21]. Soy isoflavones (genistein, daidzein, equol) are major group of phytoestrogens and the most common botanical supplement used by perimenopausal women. Other phytoestrogens include coumestrol, resveratrol, zearalenone, α-zearalanol, and biochanin A. 

## DOSE OF ESTROGEN IN HRT

The plasma levels of estrogen decline precipitously during menopause. Thus, the primary objective of HRT has often been to restore the plasma estrogen to levels observed in premenopausal women. This view should be analyzed with extreme care. Estrogens are highly lipophilic compounds, and their circulating levels may not necessarily reflect their vascular tissue level. Also, hormones are extensively bound to plasma proteins and the hormone pharmacokinetics and volume of distribution may change during menopause, particularly in the presence of kidney, liver or metabolic disease. Thus, an apparently normal dose of estrogen could lead to superphysiological plasma levels in menopausal women, and smaller doses of estrogen appear to be as effective as a traditional dose [22-24].

## ROUTE OF ADMINISTRATION OF HRT

The outcome of HRT could also vary depending on the route of administration. Because estrogens are highly lipophilic they are well absorbed when taken orally, and therefore various combinations of estrogen have been used in clinical trials, mainly as oral pills. However, the first-pass metabolism of estrogen in the liver could determine its cardiovascular effects. Estradiol is rapidly metabolized in the liver and is not very effective orally. Ethinyl estradiol is more effective orally, and estradiol benzoate is available in a slow-release form for parenteral administration. Because of their lipophilicity, estrogens are also absorbed through the skin. For example, estriol has been used as topical skin treatment to manage the effects of aging and menopause by decreasing facial wrinkles, smoothing skin, and maintaining healthy skin in the vagina and urethra. However, the vascular effects associated with the use of topical estriol preparations are understudied. Estradiol patches such as Estraderm, Vivelle-Dot, Alora, and Climara are also available and transdermal estrogens such as Estrasorb (estradiol topical emulsion) may confer more cardiovascular benefits than the oral forms. Studies compared markers for atherosclerotic vascular disease and endothelial function in healthy postmenopausal women receiving oral CEE 0.625 mg/day or transdermal estradiol gel 0.6 mg/day for 6 months. Flow-mediated brachial artery vasodilation was increased in both the oral and transdermal HRT groups, suggesting that transdermal estrogen, similar to oral estrogen, exerts a positive effect on endothelial function. On the other hand, the levels of the vascular inflammatory marker C-reactive protein (CRP) rose in the oral but not the transdermal HRT group, suggesting that oral estrogen may promote vascular inflammation and atherosclerotic vascular risk, while transdermal estrogen avoids this untoward effect and might be more favorable in reducing the risk of atherosclerotic vascular disease [25]. Similar results were observed in postmenopausal women treated with either transdermal estradiol or oral CEE; both routes included MPA. CRP levels significantly increased in the women orally administered HRT, but did not change in women treated transdermally. Also, transdermal estradiol administration was associated with an increase in the brachial-ankle pulse wave velocity, which may be related to the direct effect of E2, but no change in the brachial-ankle pulse wave was observed in women administered HRT orally. These data suggest that transdermal estradiol may reduce the risk of atherosclerosis by improving arterial stiffness [26].

Vaginal estrogen in the form of creams, tablets, or rings is effective for genitourinary symptoms, and an excellent option for nearly all postmenopausal women with the exception of breast cancer patients. Vaginal estrogen preparations can be administered long-term as their systemic absorption is low, but their potential long term vascular effects should still be considered.

## VASCULAR ESTROGEN RECEPTORS (ERS)

Changes in vascular ERs could also occur during menopause, and may influence the outcome of HRT. Vascular ERs have been identified in the mouse aorta, rat aorta, carotid, uterine and tail artery, rabbit uterine artery, bovine aorta; primate coronary and carotid artery, and human coronary and uterine artery and umbilical vein. However, a recent study has reported that estrogen affects vascular tone and ER expression in human arteries, but not in human veins; a finding that should be taken into consideration when examining the effects of estrogen on the systemic circulation [27]. ERs are expressed in virtually all components of the vascular wall including the endothelium, VSM and adventitial cells. Two ER subtypes, ERα and ERβ have been identified [3,4,28]. However, they are not impervious to genetic polymorphism which could affect the receptor binding properties. Truncated forms of ERα and ERβ have been described in many tissues, including the vascular endothelium [29,30]. Generation of a full length mature RNA transcript results in a 66 kDa ERα. ERα variants (55 and 46 kDa) have been described and the 46 kDa variant is abundant in endothelial cells [29,30]. A recent study has demonstrated sex difference in the relationship between arterial stiffness and the -401T/C or 30T/C polymorphisms in ER-α in older humans. Polymorphisms in these genes was associated with decreased brachial-ankle pulse-wave velocity in postmenopausal women, but not in men [31].

Classical ERs require hours to days to illicit an effect on gene expression, while those located in endothelial cells respond to estrogen within minutes [32]. Estrogen diffuses through the plasma membrane and forms complexes with cytosolic/nuclear ERs, which then bind to chromatin, stimulate gene transcription and induce genomic effects. Estrogen also binds to signal-generating ERs on the plasma membrane of vascular cells and induces rapid non-genomic effects. It appears that ERβ mediates most of the vascular action of estrogen, as the same vascular protection is observed in ERα knockout and wild-type mice [33]. 

Downregulation of vascular ER during menopause could take the form of decreased expression of the receptor and/or decreased affinity of the receptor to estrogen. Although our studies have shown little change in ER expression in the aorta of aging compared with adult OVX SHR [34], these observations need to be verified in other animal species as well as in human vascular tissues. Also, methylation halts the activation of gene expression in multiple systems, including the cardiovascular system. Methylation of the promoter region of the ER gene has been associated with the downregulation of the receptor. An age-related increase in ER methylation was observed in vascular tissue. This modification could inhibit the protective effects of E2 and could explain the diminished benefits of HRT observed in the postmenopausal women [35]. 

## MODULATORS OF VASCULAR ERS

The discovery of different ER subtypes and variants has increased the hopes of developing compounds that specifically target the vascular ER. Tissue-selective ER modulators (SERMs) are non-steroidal estrogenic drugs that interact with ER, but differ from estrogen in that they elicit either agonist or antagonist effects depending on the target tissue. Examples of SERMs are raloxifene, toremifene, idoxifene and tamoxifen [36]. 

SERMs may be more selective in targeting the vascular ERs. Tamoxifen positively modulates cerebrovascular tone in ovariectomized female rats, acting as an estrogen agonist during estrogen deficiency [37]. Similarly, raloxifene elicits rapid endothelium-dependent nitric oxide-mediated vasorelaxation in rat aorta that is not sensitive to either the nonselective ER antagonist ICI 182,780 or the selective ERα antagonist 1,3-bis (4-hydroxyphenyl)-4-methyl-5- (4-(2-piperidinylethoxy) phenol)-1H-pyrazole (MPP) [38]. Raloxifene also elicits long-term anti-inflammatory actions in rat aortic VSM cells *via* upregulation of ERα protein levels [39].

The Raloxifene Use for The Heart (RUTH) clinical trail examined the risk-benefit ratio of raloxifene in preventing acute coronary events and invasive breast cancer [40]. Although raloxifene reduced the risk of invasive breast cancer and vertebral fractures, it did not significantly affect the risk of coronary heart disease and was associated with increased risk of venous thromboembolism and fatal stroke. Thus the benefits of raloxifene in reducing the risks of invasive breast cancer and vertebral fracture should be weighed against the increased risks of venous thromboembolism and fatal stroke [41].

Selective ER agonists and antagonists may be useful in maximizing the vascular effects of estrogen while reducing its adverse effects on uterine and breast cancer. Diarylpropionitrile DPN is a potent ERβ agonist with a 30- to 70-fold selectivity over ERα [42]. The phytoestrogen biochanin A is also a selective ERβ agonist while RR-tetrahydrochrysene is a selective ERβ antagonist. Ethinyl estradiol is a relatively specific ERα agonist. Triarylpyrazoles such as propylpyrazole trisphenol (PPT) are approximately 400-fold more potent on ERα than ERβ [43], and MPP is an ERα selective antagonist [38]. 

## POST-RECEPTOR VASCULAR EFFECTS AND SIGNALING MECHANISMS

Possible changes in the ER post-receptor signaling mechanisms could occur during menopause, and may render the estrogen-ER interaction in vascular target tissues ineffective. Studies have shown significant effects of estrogen on modification of circulating lipoproteins, inhibition of lipoprotein oxidation, attenuation of atherosclerotic lesions, favorable modulation of homocysteine, changes in blood coagulation, and inhibition of intravascular accumulation of collagen. Estrogens may also inhibit the angiotensin converting enzyme (ACE) and renin release, leading to significant changes in the renal hemodynamics and renal control mechanisms of the blood pressure [44,45]. In OVX female rats, regular subcutaneous administration of E2 appeared to prevent an increase in ACE activity, as seen in OVX female rats treated with a subcutaneous vehicle [46].

Gender differences in vascular function have also been described [1]. Vascular contraction is greater in blood vessels of intact male than intact female rats, not different between castrated and intact males, but greater in ovariectomized (OVX) than intact females. Estrogen replacement in OVX female rats restores the vascular contraction to its level in intact females, suggesting that the gender differences in vascular contraction may involve direct effects of estrogen on the vasculature.

**Fig. (1):**
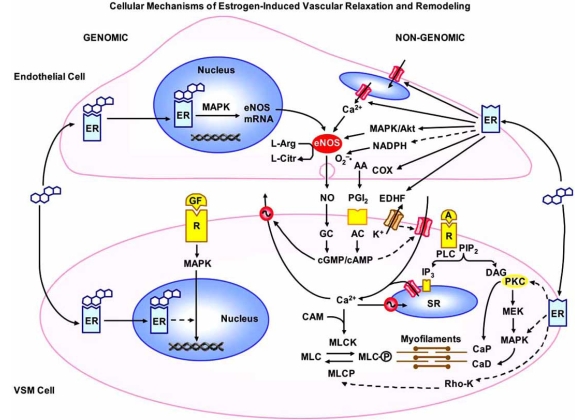
Cellular mechanisms of estrogen-Induced vascular relaxation and remodeling. In endothelial cells, estrogen binds to cytosolic/ nuclear ERs, activates MAPK, and stimulates gene transcription and eNOS expression. Estrogen also binds to surface membrane ERs and stimulates Ca^2+^ release from the endoplasmic reticulum, the MAPK/Akt pathway, eNOS activity and NO production. NO activates guanylate cyclase (GC) and increases cGMP which causes VSM relaxation by inhibiting Ca^2+^ influx and stimulating Ca^2+^ extrusion. Estrogen’s antioxidant  effects cause decreases in NADPH and O_2_^–^• production and increases in NO bioactivity. Estrogen also activates cyclooxygenases (COX) and enhances the production of PGI2, which in turn activates adenylate cyclase (AC), increases cAMP, and causes VSM relaxation by mechanisms similar to those of cGMP. ER also increases the release of EDHF, activates K+ channels and causes VSM hyperpolarization and inhibition of Ca^2+^ influx *via* Ca^2+^ channels. Estrogen also activates cytosolic/nuclear ERs in VSM and inhibits growth factor (GF)-mediated activation of MAPK, gene transcription and VSM growth. Estrogen also binds to plasma membrane ERs, decrease [Ca^2+^]i and inhibit Ca^2+^- dependent MLC phosphorylation and VSM contraction. Estrogen also inhibits PKC-, MAPK- and Rho kinase and thereby leads to decreases in the myofilament force sensitivity to [Ca^2+^]i. Dashed arrows indicate inhibition. A: agonist, R: receptor, PLC: phospholipase C, PIP2: phospatidyinositol 4,5-bisphosphate, IP3: inositol 1,4,5-trisphosphate, DAG: diacylglycerol, SR: sarcoplasmic reticulum, CAM: calmodulin, MLC: myosin light chain, MLCK: MLC kinase, MLCP: MLC phosphatase, MEK: MAPK kinase, CaP: calponin, CaD: caldesmon.

Estrogen induces both genomic and non-genomic effects in the vasculature. Nuclear ERs act as transcription factors that modulate gene expression by directly binding to DNA at specific estrogen response elements. ERs could also indirectly prevent transcription of promoters lacking estrogen response elements by interacting with nuclear transcription factors. ER transcriptional activity may be regulated by intracellular signaling pathways even in the absence of ER ligands [36]. Activation of cytosolic/nuclear ERs in endothelial cells triggers genomic effects leading to cell growth and proliferation. For example, 17β-estradiol (E2) induces the phosphorylation and activation of mitogen-activated protein kinase (MAPK) and proliferation of endothelial cells (Fig. **1**). In contrast, E2 inhibits MAPK activity and the growth and proliferation of VSM cells [47,48]. E2 could also activate plasma membrane ERs in the endothelium and VSM, initiate non-genomic effects, and cause reduction in vasoconstriction [49]. 

## ESTROGEN AND THE ENDOTHELIUM

E2-induced vasodilation is mediated to a large extent by the endothelium [50]. Estrogen induces vascular relaxation by modifying the synthesis/release/bioactivity of endothelium-derived relaxing factors such as nitric oxide (NO), prostacyclin (PGI_2_) and hyperpolarizing factor (EDHF), as well as contracting factors such as endothelin (ET-1). E2 potentiates endothelium-dependent flow-mediated vasodilation in postmenopausal women. Also, endothelium-dependent vascular relaxation is greater in female than male spontaneously hypertensive rats (SHR). A significant relationship exists between the amount of ERs and endothelium-dependent NO release. Basal release of NO, as determined by endothelium-dependent vasodilation is larger in the aorta of wild-type male mice than their ER knockout counterparts [51]. ERα appears to mediate most of the cardioprotective actions of estrogen including nongenomic vasodilation [52,53]. Estrogen treatment increases basal NO production in the aorta of mice expressing only functional ERα [54]. Also, E2-induced vascular relaxation is more pronounced in ERβ-deficient mice [55]. Additionally, selective ERα agonists improve endothelial dysfunction in blood vessels of OVX SHR [56]. On the other hand, ERβ mediates non-genomic action in caveolae, where the eNOS enzyme is localized [57,58]. 

Total NO production is greater in premenopausal women than in men [59]. Similarly, endothelial NO release is greater in the blood vessels of female than male rats. Estrogen may influence NO production by activating ER-mediated genomic pathways and up-regulating endothelial NO synthase (eNOS). E2 stimulation and ERα gene transfer into endothelial cells induces eNOS gene expression. Also, membrane-impermeant E2 binds to ER at the cell surface, stimulates non-genomic increases in [Ca^2+^]_I_, NOS activity and NO release from human endothelial cells. E2 also promotes the association of heat shock protein 90 (Hsp90) with eNOS and reduces the Ca^2+^ requirement for its activation [60]. The E2-induced [Ca^2+^]_i _causes transient translocation of eNOS from endothelial cell plasma membrane to intracellular sites close to the nucleus, while prolonged exposure to E2 induces the return of eNOS to the plasma membrane for its full activation [3]. E2 also induces the phosphorylation/activation of eNOS by increasing the activity of MAPK (ERK1/2) or the phosphatidylinositol-3 (PI_3_)-kinase-Akt pathway [61]. 

Estrogen has antioxidant properties that could affect NO bioavailability [62]. In OVX female rats, increased blood pressure is associated with lower plasma antioxidant levels and increased plasma lipoperoxides and vascular free radicals. E2 replacement prevents these effects. Also, the amount of superoxide (O_2_^–^•) is greater in blood vessels of male than female rats. Furthermore, E2 inhibits NADPH oxidase expression and the generation of O_2_^–^• and peroxynitrite (ONOO^–^), thereby enhancing NO bioactivity.

PGI_2_ is produced from the metabolism of arachidonic acid by cyclooxygenases (COX). The COX inhibitor indomethacin decreases endothelium-dependent vascular relaxation, and gender differences in indomethacin-sensitive vascular relaxation have been attributed to differences in COX products [63]. Also, E2 causes upregulation of COX-1 expression and PGI_2_ synthesis in endothelial cells. Estrogen also increases urinary excretion of COX-2–derived PGI_2_ metabolites in ERβ but not ERα knock-out mice [64].

Acetylcholine (Ach)-induced hyperpolarization and relaxation of mesenteric arteries are reduced in males and OVX females as compared to intact female rats, and the difference is eliminated by K^+^ channel blockers. Also, the hyperpolarizing response to Ach is improved in E2-replaced OVX female rats, confirming that estrogen-deficient states attenuate vascular relaxation by endothelium-derived hyperpolarizing factor (EDHF). 

E2 modulates ET-1 expression and release in human endothelial cells [65]. Also, prolonged treatment of endothelial cells with E2 inhibits ET-1 production in response to serum, tumor necrosis factor-α, transforming growth factor β1 and AngII [66]. Additionally, ET-1 release from endothelial cells is less in female than male SHR. ET-1 activates endothelial ET_B1 _receptor and causes the release of relaxing factors that promote vascular dilation. ET-1 also stimulates ET_A_ and ET_B2_ receptors and causes VSM contraction. In mesenteric arteries of deoxycorticosterone acetate (DOCA)-salt hypertensive rats, contraction to ET-1 is greater in males than females. Ovariectomy is associated with increased ET-1 and ET_B _receptor mRNA in mesenteric arteries, and E2 replacement reverses these changes. Also, the ET_B_ agonist IRL-1620 induces less vasoconstriction in intact compared with OVX females, and E2 replacement decreases IRL-1620-induced vasoconstriction in OVX females. These data suggest that ovarian hormones attenuate ET-1/ET_B _receptor expression and their vascular responses in DOCA-salt hypertensive rats [67].

## ESTROGEN AND MECHANISMS OF VSM CONTRACTION

Estrogen causes relatively rapid relaxation in endothelium-denuded blood vessels [49]. The acute inhibitory effects of E2 on vascular contraction *in vitro* are observed at ambient concentrations in the micro-molar range, which exceed the physiological pico-molar concentrations in the plasma, and are believed to involve non-genomic effects on the mechanisms of VSM contraction. The greater plasma estrogen levels in females may explain the reduced vascular contraction in females compared with males. Also, ERs are more abundant in arteries of females compared with those of males [68]. E2 also induces downregulation of vascular angiotensin AT_1_ receptor mRNA and protein [46,69]. The gender differences in vascular contraction could also be due to differences in the signaling mechanisms of VSM contraction downstream from receptor activation.

VSM contraction is triggered by increases in [Ca^2+^]_i_ due to Ca^2+^ release from the sarcoplasmic reticulum and Ca^2+^ entry from the extracellular space. Activation of myosin light chain (MLC) kinase, Rho kinase and MAPK also contribute to VSM contraction. Also, the agonist-receptor interaction is coupled to increased production of diacylglycerol, which activates protein kinase C (PKC) [70].

In isolated VSM cells incubated in the presence of external Ca^2+^, phenylephrine (Phe) causes an initial peak in [Ca^2+^]_i_ mainly due to Ca^2+^ release from the intracellular stores, and a maintained [Ca^2+^]_i_ due to Ca^2+^ entry from the extracellular space. In Ca^2+^-free solution, Phe or caffeine causes transient cell contraction and [Ca^2+^]_i_ that are not different between intact and gonadectomized male and female rats, suggesting that the gender differences in VSM contraction do not involve Ca^2+^ release from the intracellular stores. In contrast, the maintained Phe-induced VSM [Ca^2+^]_i_ is greater in intact male than female rats, suggesting gender differences in the Ca^2+^ entry mechanism of VSM contraction. The maintained Phe-induced [Ca^2+^]_i_ is greater in OVX than intact females, but not different between E2-replaced OVX and intact females, or between castrated and intact males, suggesting that the gender differences are likely related to estrogen [71]. The cause of the gender differences in Ca^2+^ entry may be related to the plasmalemmal density and/or permeability of VSM Ca^2+^ channels.

Estrogen causes rapid relaxation of isolated vessels possibly through an effect on Ca^2+^ mobilization and fluxes. E2 does not inhibit caffeine- or carbachol-induced VSM contraction or [Ca^2+^]_i_ in Ca^2+^-free solution, suggesting that it does not inhibit intracellular Ca^2+ ^release. On the other hand, E2 inhibits maintained agonist- and KCl-induced contraction, Ca^2+^ influx and [Ca^2+^]_I_, suggesting inhibition of Ca^2+^ entry through Ca^2+ ^channels [72]. 

E2 activates BK_Ca_ channels in coronary VSM, leading to hyperpolarization and decreased Ca^2+^ entry through voltage-gated channels. In human coronary artery smooth muscle cells ER( may mediate acute BK_Ca_ channel stimulation by E2. When the ERα  mRNA was abolished with an ERα plasmid, the stimulatory effect of E2 on the BK_Ca _was fully eliminated. Because this effect occurred within minutes of the introduction of the plamsid, this is thought to be a non-genomic effect of E2 [73]. However, a direct effect of estrogen on Ca^2+^ channels has also been suggested. Estrogen may also decrease [Ca^2+^]_i_ by stimulating Ca^2+^ extrusion *via* plasmalemmal Ca^2+^ pump [74].

The gender differences in vascular contraction could also be due to differences in the expression/activity of PKC in VSM. Like Phe, phorbol esters, which activate PKC, produce greater contraction in isolated vessels of intact male than female rats, suggesting gender differences in the PKC-mediated pathway of VSM contraction. PKC is a family of several isoforms that have different substrates, functions and subcellular distributions. Immunoblot analysis in VSM of intact male rats has shown significant amounts of α-, δ- and z-PKC, and both Phe and phorbol esters cause activation and redistribution of α- and δ-PKC. The amount and the Phe- and phorbol ester-induced redistribution of α- and δ-PKC are less in intact female as compared to male rats, and the gender differences in VSM contraction have been related to underlying changes in the amount/activity of α-, δ- and z-PKC [75].

The Phe- and phorbol ester-induced VSM contraction and PKC activity are not different between castrated and intact male rats, but greater in OVX than intact females and E2-replaced OVX female rats, suggesting an effect of estrogen on the expression/activity of PKC. A genomic action of estrogen on PKC expression in VSM might well underlie the reduction in vascular contraction and PKC activity in female rats compared with males. However, additional non-genomic effects of sex hormones on the PKC molecule or its lipid co-factors or other protein kinases upstream from PKC cannot be excluded. 

The small GTPase RhoA has been implicated in focal adhesions, and the downstream effector Rho-kinase plays a role in the regulation of force and actomyosin crossbridge cycling in VSM [76,77]. Rho-kinase may inhibit MLC phosphatase leading to increased MLC phosphorylation and enhancement of VSM contraction. Rho-kinase may also stimulate VSM cell proliferation and migration. Abnormal activation of the Rho-kinase pathway may play a role in hypertension and other vascular diseases, and Rho-kinase inhibitors, such as fasudil, may be beneficial in CVD [78].

The expression of Rho-kinase is mediated by the PKC/NF-κB pathway, which is inhibited by estrogen [79]. In a study examining gender differences in basilar artery diameter the Rho-kinase inhibitor Y-27632 was 3-fold more potent as vasodilator in males than females. Expression of RhoA and Rho-kinase did not differ between males and females. In OVX rats, vasodilator responses to the Rho kinase inhibitor Y-27632 resembled responses in males, and treatment of OVX rats with E2 normalized the vasodilator effects of Y-27632 to those observed in intact females. These data suggest that vascular Rho kinase function is suppressed in females possibly due to estrogen effects [80].

## ESTROGEN, THE CYTOSKELETON AND EXTRACELLULAR MATRIX (ECM).

 Estrogen also plays a role in the regulation of the cytoskeleton, extracellular matrix and vascular remodeling. ERα interacts with the G protein Gα 13 to induce activation of the RhoA/Rho-kinase pathway and phosphorylation of the actin-regulatory protein moesin, leading to remodeling of the actin cytoskeleton and endothelial cell migration [81]. Interendothelial junctions are important regulators of endothelial cell functions such as migration, proliferation, and angiogenesis. E2 regulates these functions *in vivo* and *in vitro* and increases endothelial monolayer permeability by decreasing monolayer integrity and intercellular adhesion. A recent study examined whether E2 affects endothelial cell adhesion-dependent functions by targeting the adherens junction complex. It was found that E2 increases uterine microvascular endothelial cell monolayer permeability and transiently redistributes inter-endothelial junction-forming proteins. Concomitantly, adherens junction proteins are disconnected from the cytoskeleton and α-catenin, which links VE-cadherin to the cytoskeleton, is redistributed from the membrane and the adherens junction complex. Also, E2 increased tyrosine phosphorylation of the adherens junction complex, and similar effects could be provoked by non-cell membrane-permeable 17(-estradiol-BSA. Additionally, E2 treatment enhanced the angiogenic effect of vascular endothelial growth factor in an *in vitro* angiogenesis model, possibly due to the adherens junction disruption. Co-treatment with the Src-family kinase inhibitor PP2 prevented the redistribution and phosphorylation of the adherens junction proteins. It was concluded that adherens junctions in endothelial cells are a downstream target of membrane-associated E2 signaling, possibly through Src-family kinases [82]. Mitochondria may have physical effects on the cytoskeleton including changes in tension, and some of these effects may be mediated by estrogen. For example, transmission electron microscopy showed an increase in mitochondria size of MCF7 cells treated with E2. The E2-induced mitochondrial enlargement is associated with stimulation of ROS, which in turn affect the elasticity of the actin network [83].

E2 enhances endothelial cell interaction with extracellular matrix proteins *via* an increase in integrin expression and function [84]. Also, CEE inhibits aortic connective tissue remodeling in female monkeys [17]. Matrix metalloproteinases (MMPs) and tissue inhibitors of MMPs play a role in the degradation of extracellular matrix proteins and vascular remodeling during atherosclerotic progression, and E2 has been shown to enhance release of MMP-2 from cultured human VSM cells [85]. In a more recent study, MMP levels did not increase when transdermal E2 and MPA were administered to postmenopausal women. However, E2 and MPA treatment was associated with decreased carotid intima media thickness, and reduced levels of circulating vascular inflammatory markers, including intercellular adhesion molecule, vascular cell adhesion molecule, E-selectin and monocyte chemoattractant protein [86].

## ESTROGEN AND VASCULAR INFLAMMATION.

Low-grade inflammation has been implicated in CVD, and estrogen may affect the course of vascular disease by affecting vascular inflammation. In aortic segments of OVX mice, estrogen attenuates vascular expression of inflammation-associated genes and adhesion of monocytes to endothelial cells [87]. Also, E2 *via* an ERα-mediated pathway blocks Interferon-γ induced CD40 and CD40L protein expression and prevents neutrophil adhesion on porcine aortic endothelial cells [88]. ERβ also appears to mediate anti-inflammatory effects of estrogen. TNF-α-induced mRNA expression of inflammatory mediators in rat aortic smooth muscle cells is attenuated when the cells are treated with E2 or the ERβ-selective agonist, diarylpropiolnitrile (DPN) [89]. In estrogen-depleted aging OVX rats serum levels of the pro-inflammatory cytokine TNF-α are higher and the sensitivity of mesenteric arteries to Phe vasoconstriction is greater compared with estrogen-replaced animals. Also, *in vivo* treatment with the TNF-α inhibitor etanercept or estrogen replacement is associated with reduced Phe constriction of mesenteric arteries and decreased vascular expression of AngII, angiotensin-converting enzyme and AT_1_ receptor. These data suggest that upregulation of TNF-α during estrogen deficiency may contribute to enhanced vasoconstriction by altering the vascular AngII system [90]. However, the effects of estrogen on the inflammatory process may vary depending on the estrogen preparation used. For example, in healthy postmenopausal women receiving HRT, the levels of the vascular inflammatory marker CRP rose in the oral but not the transdermal HRT group, suggesting that oral estrogen may promote acute inflammation, while transdermal estrogen is devoid of these effects [25].

## ESTROGEN AND VASCULAR FUNCTION AT MENOPAUSE

Several biological and physiological changes occur at menopause, partially due to the significant decrease in plasma estrogen levels to < 100 pmol/L. Our data in the aging OVX SHR model of postmenopausal hypertension have shown decreased E2-induced vascular relaxation [34]. Although little change in vascular ER expression was observed in aging compared with adult OVX SHR, possible age-related decreases in the affinity of vascular ER to estrogen or reduction in the post-ER signaling mechanisms of endothelium-dependent vasodilation and inhibition of the mechanisms of VSM contraction may occur. One consequence of decreased endothelial function associated with aging is enhanced reactivity to vasoconstrictors. For example, the role of K^+ ^channels as modulators of endothelium-dependent relaxation decreases with age in rats, and the vasodilator responses become exclusively mediated by NO, while the EDHF responses diminish. Also, the expression of prostaglandin H synthase 1 and prostacyclin synthase increases with age. Additionally, the leakage of O_2_^–^• from mitochondria is believed to increase with age, and the role of mitochondria in vascular aging is gaining attention [91]. ERs have been found in mitochondria, especially in the cerebrovascular region and their localization increases the capacity for oxidative phosphorylation, thereby suppressing the damage of ROS and increasing the efficiency of energy production [92]. These findings are important as they highlight the significance of timing of HRT in relation to the onset of menopause and subject’s age.

## SUBJECT’S AGE AND TIMING OF HRT

The lack of beneficial vascular effects of HRT in post-menpausal women could be related to the subject age. The HERS clinical trial was performed on postmenopausal women at advanced age (66.7 years) close in range to average age of enrollment in the WHI which was 62.5 years, or 12 years post-menopause. The Estrogen Replacement and Atherosclerosis (ERA) trial evaluated the flow-mediated vasodilation of the brachial artery in postmenopausal women (mean age 65.8 years) with established coronary atherosclerosis being treated with combined E2 and MPA HRT, unopposed E2, and a placebo. No significant statistical difference in brachial flow-mediated vasodilation was found between the three groups. The absence of an effect on brachial endothelial function in women of this advanced age may explain the lack of benefit of E2 in secondary prevention of coronary heart disease [93]. Because the vascular expression or responsiveness of ERs may decline with advanced age HRT is expected to be more beneficial if started early in menopause rather than in older postmenopausal women. This is supported by a macaque atherosclerosis study in which OVX monkeys were placed on a high cholesterol diet in the absence or presence of HRT. Those placed on HRT at the time of surgical menopause had a 70% reduction in coronary atherosclerosis. In contrast, when HRT was delayed 2 years, the beneficial effect was lost [94]. Another study has shown that the administration of E2 in postmenopausal women, ranging from 45-72 years of age, only mediated improvements in flow-mediated vasodilation in the younger sub-population, under age 50. However, in all subjects, plasma concentrations of nitrite/nitrate were increased 7.5-fold [95]. In further support of the notion that age affects the results of HRT, significant decreases in flow-mediated dilation in postmenopausal women receiving short-term transdermal E2 were observed in those less than 60 years of age, but for those greater than age 60, no alterations in their flow-mediated dilation response were observed [96]. Interestingly, further sub-group analysis of data from the WHI study showed beneficial cardiovascular effect of HRT in women 50 to 59 years of age, although the data are under-powered to achieve statistical significance, based on the sub-group size [97,98]. Recent clinical trials appear to address some of these concerns. The Kronos Early Estrogen Prevention Study (KEEPS), which began in mid-2005, is a randomized controlled multicenter 5-year clinical trial that will evaluate the effectiveness of low dose CEE, and weekly transdermal estradiol (both in combination with cyclic oral, micronized progesterone) in preventing progression of carotid intimal medial thickness and the accrual of coronary calcium in early menopausal women (aged 42-58 years) who are within 36 months of their final menstrual period, and will examine surrogate end points as well as risk factors for atherosclerosis [99].

## HRT AND PREEXISTING CARDIOVASCULAR CONDITION

HRT could be more effective in reducing the incidence or the development of CVD rather than treatment of preexisting CVD. In effect, the HERS clinical trial enrolled women with pre-established CVD. Also, the subjects enlisted in the ERA and RUTH clinical trials were at high risk of coronary heart disease. The Women's Angiographic Vitamin and Estrogen (WAVE) clinical trial demonstrated that postmenopausal subjects with coronary disease did not experience any improvement in cardiovascular function as measured by flow-mediated vasodilation, when administered HRT or antioxidant vitamins for approximately three years [100]. The number of ER in atherosclerotic plaques is significantly reduced compared to those found in normal segments of human coronary arteries [51]. As described above, beneficial effects of estrogen in atherogenesis include a decrease in LDL oxidation and binding as well as a decrease in smooth muscle cell proliferation. Also, estrogen decreases cell adhesion molecules, macrophage accumulation and monocyte adhesion. Additional beneficial estrogen effects include an increase in endothelial function and vasodilation. Thus HRT given early during perimenopause may decrease the development/ progression of fibrous cap and atherogenic plaque and reduce lesion development. Adversely, in already established atherogenic plaques estrogen may increase inflammation, MMP expression and neovascularization leading to increased lesion progression, plaque instability and rupture/hemorrhage, respectively. This may explain the reduced vascular benefits of HRT in postmenopausal women with preexisting CVD and further underscore the importance of HRT timing.

## ROLE OF PROGESTERONE

The hormone combination used could also affect the outcome of HRT in postmenopausal CVD, and progesterone may alter the vascular effects of estrogen. Plasma progesterone ranges from 1.5 nmol/L in the follicular phase to 35 nmol/L in the mid-luteal phase, and significantly decreases to < 1 nmol/L during menopause. Also, progesterone receptors have been identified in the endothelium and VSM of vascular beds of the mouse, rat, rabbit, primates as well as human. In addition to natural progesterone several synthetic progestins are available including medroxyprogesterone acetate (MPA), norethisterone, norgestimate, 3-keto-desogestrel, levonorgestrel and gestodene. Also, a highly publicized progesterone antagonist is RU486 (Mifepristone).

Similar to E2, progesterone has anti-atherosclerotic effects, decreases LDL and increases HDL. Also, progesterone stimulates eNOS expression, NO production and NO-mediated relaxation in rat aorta, porcine coronary artery and ovine uterine artery. It has been shown that subcutaneous administration of natural progesterone, not MPA, restores the endothelium-dependent attenuation of contractile responses to phenylephrine in the mesenteric arteries OVX rats [101]; therefore the use of synthetic progesterone in research studies should be further evaluated. Progesterone may also cause direct nongenomic COX activation and increase vascular PGI_2_ production. Progesterone inhibits VSM proliferation/migration and facilitates the inhibitory effects of estrogen. Progestins may also modify the effects of E2 on vascular contraction or cause rapid relaxation in endothelium-denuded blood vessels [49]. The effects of progesterone on VSM [Ca^2+^]_i_ are not clear, but acute application of progesterone decreases Ca^2+^ influx and [Ca^2+^]_i_ in rabbit and porcine coronary VSM [72]. Also, progesterone inhibits phorbol ester-induced contraction, PKC activation and translocation in VSM, an effect possibly mediated by increasing cAMP levels in VSM [102].

We should note that the combined endothelium-dependent and endothelium-independent vasorelaxant effects of progesterone are less than those of estrogen. Also, progesterone may affect vascular relaxation in a tissue-specific fashion. For example, progesterone counteracts the stimulatory effects of estrogen on NO production and vascular relaxation in canine coronary artery. Also, progesterone antagonizes the vasoprotective effect of estrogen on antioxidant enzyme expression and function and enhances NADPH oxidase activity and the production of reactive oxygen species in OVX mice [103]. Additionally, progesterone causes upregulation of vascular angiotensin AT_1_ receptor mRNA and protein, an effect that could enhance vasoconstriction [46,69]. Although the anti-inflammatory effects of estrogen would attenuate ischemic brain injury, this vasoprotective benefit may be diminished in the presence of progestins [104]. Similarly, the addition of progesterone to E2 treatment was shown to abolish the benefits of E2 alone on increases in HDL-cholesterol in postmenopausal women with hypercholesterolemia and hypertension [105]. Also, in OVX female rats LPS induces cerebrovascular iNOS and COX-2, and this effect is decreased in E2 replaced rats. In contrast, treatment of OVX females with either MPA or progesterone exacerbated the cerebrovascular inflammatory response to LPS. Additionally, CEE alone, but not in combination with MPA, inhibit aortic connective tissue remodeling after plasma lipid lowering in female monkeys [17]. Furthermore, MPA appears to antagonize the inhibitory effects of CEE on coronary artery atherosclerosis [106].

## ROLE OF ANDROGENS

Endogenous testosterone levels may also affect the outcome of HRT in postmenopausal women. Total plasma testosterone in adult females range between 1.0 to 1.5 nmol/L. Although plasma testosterone may decline during menopause to 0.3-0.5 nmol/L, the androgen/estrogen ratio is increased in postmenopausal women. Androgen receptors have been identified in the mouse, rat, rabbit and bovine aorta as well as in primate coronary artery and human internal mammary artery [107]. In addition to natural testosterone and dihydrotestosterone, there exist testosterone precursors and synthetic androgens, including androstenedione, dehydroepiandrosterone and cyproterone acetate. Androgen receptor antagonists such as flutamide, hydroxyflutamide, and bicalutamide are also available.

Testosterone is anti-atherosclerotic and may exert beneficial effects on the development of coronary atherosclerosis. It is likely that some of the effects of testosterone are due to its conversion into estradiol by aromatase and activation of ER. However, a direct role of androgens on the vascular wall has been proposed. Testosterone induces direct vasodilation in some vascular preparations [49]. The non-genomic vascular effects of testosterone involve both endothelium-dependent mechanisms and direct effects on VSM [107]. In a recent study, testosterone was found to be positively correlated to flow-mediated dilation in the brachial artery of postmenopausal women; where women with greater concentrations of testosterone had greater dilation compared to those with decreased concentrations [108]. When testosterone is acutely administered in canine coronary vessels it induces NO-mediated vasodilation. Also, dehydroepiandrosterone stimulates NO production in human endothelial cells by enhancing the expression/stabilization of eNOS. In SHR blood vessels, testosterone appears to release EDHF and causes VSM hyperpolarization by a mechanism involving large conductance Ca^2+^-dependent K^+^ channels (BK_Ca_) [109]. Testosterone also modulates VSM cell proliferation in a dose-dependent manner, with low concentrations stimulating and high concentrations inhibiting DNA synthesis and VSM growth [1]. A portion of testosterone-induced vasorelaxation is endothelium-independent and may involve ATP-sensitive K^+^ channels (K_ATP_) in VSM [110]. Studies suggest that testosterone induces vasorelaxation by inhibiting Ca^2+^ influx and decreasing [Ca^2+^]_i_ in VSM [72]. The vasorelaxant effect of testosterone is attenuated by K^+^ channel blockers, suggesting that stimulation of K^+^ conductance is involved in the inhibitory effects of testosterone on VSM [Ca^2+^]_I_. Also, dihydrotestosterone decreases TNF-α and LPS-induced inflammatory response in human endothelial cells [111].

Some studies suggest the use of androgen supplements to enhance libido and social well-being in menopausal women. However, androgens may have adverse effects on the rennin-angiotensin system and the kidney and could play a role in the development of some forms of hypertension [2,112]. Specific androgen antagonists may block the harmful effect of androgens on the kidney while enhancing their beneficial vascular effects.

## PERSPECTIVE

The greater incidence of CVD in men and postmenopausal women compared with premenopausal women is related to effects of sex hormones on vascular function. Although experimental studies suggest both genomic and non-genomic beneficial effects of estrogen on the endothelium, VSM and adventitial cells, these effects did not translate into vascular benefits in clinical trials of HRT in postmenopausal women. The lack of vascular benefits of HRT in postmenopausal women appears to involve several factors related to the hormone used, the vascular ERs and the subjects’ age and preexisting cardiovascular condition. 

New directions of HRT research should focus on compounds that specifically target the vascular ERs. For example, selective ERα agonists improve endothelial dysfunction in estrogen-deficient rats [56]. Also, SERMs could be more specific in targeting the vascular ERs with little undesirable growth promoting effects on breast cancer. Natural hormones that avoid first-pass metabolism in the liver could be more effective HRT. Likewise, transdermal estrogen may be more beneficial than oral estrogen in enhancing vascular relaxation, while reducing vascular inflammation. Estradiol metabolism may also determine its cardiovascular effects, and nonfeminizing estradiol metabolites may confer cardiovascular protection in both genders. Furthermore, phytoestrogens and “hormone bioidenticals” may provide a more natural dietary source of estrogen replacement than synthesized compounds.

The ER subtypes, distribution and function in vascular cells also need to be further examined. ER variants and truncated forms of ERα and ERβ have been identified in many tissues including the endothelium and VSM and may alter the vascular effects of estrogen [29,30]. Also, the subcellular distribution of ERs at the cell membrane, cytosol and nuclear compartment could determine the net genomic and nongenomic vascular effects of estrogen. Additionally, ERs are phosphoproteins, and mutations in phosphorylation sites may affect their transactivation capacity. Differences in ER subtypes, subcellular distribution and post-receptor signaling mechanisms may explain why estrogen enhances endothelial cell growth, but inhibits VSM proliferation. For example, in mice with genetic ERα deletion, the cardioprotective role of estrogen in ischemia/reperfusion injury is lost [113,114]. On the other hand, genetic deletion of ERβ results in the development of hypertension [28].

In addition to nuclear ERs that mediate the classic genomic effects of estrogen, estrogen binds to cell membrane ERs and induces rapid nongenomic effects on the mechanisms of vascular relaxation/contraction. These effects include increased endothelium-derived vasodilators as well as inhibition of VSM [Ca^2+^]_I_, PKC and Rho kinase. Other signaling pathways such as MLC kinase and phosphatase, and tyrosine kinase could regulate VSM contraction, and the changes in the expression/activity of these protein kinases and phosphatases with gender and gonadal hormones need to be further examined.

The lack of vascular benefits of HRT in postmenopausal women could also be related to its timing/duration and the subjects’ age or preexisting cardiovascular condition. Studies have shown that ovariectomy augments hypertension in aging female Dahl salt-sensitive rats [115]. Also, age-related reduction in ER-mediated mechanisms of vascular relaxation has been observed in blood vessels of female SHR, providing evidence that the vascular effects of estrogen change with aging [34]. Studies such as the KEEPS clinical trial are needed to clarify whether using HRT in women within the menopausal transition can provide cardiovascular protection. Also, the type, dose, timing/duration of estrogen treatment should be customized depending on the subject’s age and preexisting cardiovascular condition.

Similar to estrogen, progesterone appears to confer cardiovascular effects and could modify the vascular effects of estrogen, and therefore, the benefits/risks of including progesterone in the HRT combination should be further examined. Although androgens may be involved in some forms of hypertension by up-regulating the renal renin-angiotensin system, data suggest an effect of testosterone on the vascular control mechanisms of blood pressure. The effects of testosterone on the mechanisms of vascular relaxation/contraction warrant further examination of its role in coronary artery disease and hypertension. However, it is important to note that sex steroids have different sexual effects, and their vascular effects may be different in males and females. Gender differences in the inhibitory effects of estrogen on vascular contraction have also been observed [116]. Although modulators of the effects of progesterone and testosterone may be beneficial in postmenopausal women, steroid hormones have the same pregnenolone origin, and blocking one pathway may shift the hormonal balance. Thus, the relative vascular benefits of sex hormones should always be weighed against potential vascular risks of HRT in postmenopausal women.

## Figures and Tables

**Table 1.** Summary of Representative HRT Clinical Trials in Postmenopausal Women

Trial	HERS	HERS-II	WHI	ERA	RUTH	KEEPS
Objective	Whether HRT reduces CHD events in women with preexisting coronary disease	Observational follow-up to HERS	Whether HRT reduces CHD events in healthy postmenopausal women	Whether HRT reduces progression of coronary atherosclerosis, assessed by angiography	Whether Raloxifene lowers the risk of coronary events & invasive breast cancer	Whether HRT prevents progression of carotid intimal medial thickness and accrual of coronary calcium
Design	Double blind randomized clinical trial (DB RCT) of secondary prevention	DB RCT of secondary prevention	RCT of primary prevention	DB RCT	DB RCT	DB RCT of secondary prevention
Cohort Criteria	Women with preexisting CAD	Women with preexisting CAD	Healthy women (no history of CAD)	Women with documented CHD; half had previous MI	Half had CHD; remainder had multiple CHD risk factors	Women within 36 months of their final menstrual period
Age (yr)	66.7	66.7	63 (50-79)	65.8	≥55 (mean 68, 39% >70)	42-58
HRT Used	Oral 0.625 mg CEE + 2.5 mg MPA/day	Open-label HRT prescribed at personal physicians' discretion	Oral 0.625 mg CEE+ 2.5 mg MPA/day or oral 0.625 mg CEE/day	Oral 0.625 mg CEE + 2.5 mg MPA/day or oral 0.625 mg CEE/day	60 mg/day Raloxifene	0.45 mg/day oral CEE; 50 µg/day weekly transdermal estradiol, both with cyclic oral micronized progesterone (200 mg/day, 12 days/mth)
Number Enrolled	2,763	2,321 consented to follow-up	16,608 with intact uterus received CEE+MPA; 10,739 without uterus received CEE	309	10,101 at 187 sites in 26 countries	720
Study Duration	4.1 years (1993 - 1998)	Additional 2.7 years	Enrollment began in 1993; designed as 15 year trial	3.25 years	Enrollment began June 1998	5 years (2005-2010)
Outcome	172 MI's and coronary deaths in HRT group, 176 in placebo; increase in events in first year; beneficial impact after 2 years	No benefit; increase in deep venous thrombosis (DVT) and pulmonary embolism (PE) (RR 2.89); total events increased; 261 deaths in HRT group, 239 in placebo	Stopped CEE/MPA arm after 5.2 of planned 8.5 yrs; 29% increase in MI's, 41% increase in stroke, doubling of DVT and PE, 26% increase in breast cancer, decrease in colorectal cancer and fractures; thrombotic risk greatest in first year; CEE alone found no difference in MI's, increase in stroke, DVT and PE's, uncertain effect on breast cancer and a decrease in fractures	Angiography detected no difference in disease progression despite a favorable effect on HDL (increase) and LDL (decrease)	Raloxifene reduced the risk of invasive breast cancer and vertebral fractures, but did not significantly affect the risk of CHD, and was associated with increased risk of venous thromboembolism and fatal stroke	In progress

## ABBREVIATIONS

AngII =: Angiotensin II
CVD =: Cardiovascular disease
cAMP =: Cyclic adenosine monophosphate
COX =: Cyclooxygenase
DOCA =: Deoxycorticosetrone acetate
E2 =: 17β-estradiol
EDHF =: Endothelium-derived hyperpolarizing factor
ER =: Estrogen receptor
ET-1 =: Endothelin
HRT =: Hormone replacement therapy
MAPK =: Mitogen-activated protein kinase
MLC =: Myosin light chain
NO =: Nitric oxide
NOS =: NO synthase
O_2_^–^•: Superoxide anion
OVX =: Ovariectomized
PGI_2_ =: Prostacyclin
Phe =: Phenylephrine
PKC =: Protein kinase C
SHR =: Spontaneously hypertensive rat
VSM =: Vascular smooth muscle

## References

[1] Orshal JM, Khalil RA (2004). Gender, sex hormones, and vascular tone. Am J Physiol Regul Integr Comp Physiol.

[2] Reckelhoff JF (2005). Sex steroids, cardiovascular disease, and hypertension: unanswered questions and some speculations. Hypertension.

[3] Mendelsohn ME (2002). Genomic and nongenomic effects of estrogen in the vasculature. Am J Cardiol.

[4] Mendelsohn ME, Karas RH (2005). Molecular and cellular basis of cardiovascular gender differences. Science.

[5] Jazbutyte V, Arias-Loza PA, Hu K (2008). Ligand-dependent activation of ERbeta lowers blood pressure and attenuates cardiac hypertrophy in ovariectomized spontaneously hypertensive rats. Cardiovasc Res.

[6] Rosano GM, Sarrel PM, Poole-Wilson PA, Collins P (1993). Beneficial effect of oestrogen on exercise-induced myocardial ischaemia in women with coronary artery disease. Lancet.

[7] Grodstein F, Stampfer MJ, Manson JE (1996). Postmenopausal estrogen and progestin use and the risk of cardiovascular disease. N Engl J Med.

[8] Dubey RK, Imthurn B, Zacharia LC, Jackson EK (2004). Hormone replacement therapy and cardiovascular disease: what went wrong and where do we go from here?. Hypertension.

[9] Hulley S, Grady D, Bush T (1998). Randomized trial of estrogen plus progestin for secondary prevention of coronary heart disease in postmenopausal women Heart and Estrogen/progestin Replacement Study (HERS) Research Group. JAMA.

[10] Grady D, Herrington D, Bittner V, HERS Research Group (2002). Cardiovascular disease outcomes during 6.8 years of hormone therapy: Heart and Estrogen/progestin Replacement Study follow-up (HERS II). JAMA.

[11] Rossouw JE, Anderson GL, Prentice RL (2002). Writing Group for the Women's Health Initiative Investigators. Risks and benefits of estrogen plus progestin in healthy postmenopausal women: principal results From the Women's Health Initiative randomized controlled trial. JAMA.

[12] Grimes DA, Lobo RA (2002). Perspectives on the Women's Health Initiative trial of hormone replacement therapy. Obstet Gynecol.

[13] Koledova VV, Khalil RA (2007). Sex hormone replacement therapy and modulation of vascular function in cardiovascular disease. Expert Rev Cardiovasc Ther.

[14] Vitale C, Fini M, Leonardo F (2001). Effect of estradiol valerate alone or in association with cyproterone acetate upon vascular function of postmenopausal women at increased risk for cardiovascular disease. Maturitas.

[15] Seeger H, Petersen G, Schulte-Wintrop E, Teichmann AT, Mueck AO (2002). Effect of two oral contraceptives containing ethinylestradiol and levonorgestrel on serum and urinary surrogate markers of endothelial function. Int J Clin Pharmacol Ther.

[16] Johnson JV, Lowell J, Badger GJ, Rosing J, Tchaikovski S, Cushman M (2008). Effects of oral and transdermal hormonal contraception on vascular risk markers: a randomized controlled trial. Obstet Gynecol.

[17] Register TC, Adams MR, Golden DL, Clarkson TB (1998). Conjugated equine estrogens alone, but not in combination with medroxyprogesterone acetate, inhibit aortic connective tissue remodeling after plasma lipid lowering in female monkeys. Arterioscler Thromb Vasc Biol.

[18] Head KA (1998). Estriol: safety and efficacy. Altern Med Rev.

[19] Dubey RK, Jackson EK, Gillespie DG, Zacharia LC, Imthurn B, Keller PJ (2000). Clinically used estrogens differentially inhibit human aortic smooth muscle cell growth and mitogen-activated protein kinase activity. Arterioscler Thromb Vasc Biol.

[20] Vera R, Jiménez R, Lodi F (2007). Genistein restores caveolin-1 and AT-1 receptor expression and vascular function in large vessels of ovariectomized hypertensive rats. Menopause.

[21] Si H, Liu D (2008). Genistein, a soy phytoestrogen, upregulates the expression of human endothelial nitric oxide synthase and lowers blood pressure in spontaneously hypertensive rats. J Nutr.

[22] Sanada M, Higashi Y, Nakagawa K (2003). A comparison of low-dose and standard-dose oral estrogen on forearm endothelial function in early postmenopausal women. J Clin Endocrinol Metab.

[23] Wakatsuki A, Ikenoue N, Shinohara K, Watanabe K, Fukaya T (2004). Effect of lower dosage of oral conjugated equine estrogen on inflammatory markers and endothelial function in healthy postmenopausal women. Arterioscler Thromb Vasc Biol.

[24] Mueck AO, Genazzani AR, Samsioe G, Vukovic-Wysocki I, Seeger H (2007). Low-dose continuous combinations of hormone therapy and biochemical surrogate markers for vascular tone and inflammation: transdermal versus oral application. Menopause.

[25] Ho JY, Chen MJ, Sheu WH (2006). Differential effects of oral conjugated equine estrogen and transdermal estrogen on atherosclerotic vascular disease risk markers and endothelial function in healthy postmenopausal women. Hum Reprod.

[26] Sumino H, Ichikawa S, Kasama S (2006). Different effects of oral conjugated estrogen and transdermal estradiol on arterial stiff-ness and vascular inflammatory markers in postmenopausal women. Atherosclerosis.

[27] Haas E, Meyer MR, Schurr U (2007). Differential effects of 17beta-estradiol on function and expression of estrogen receptor alpha, estrogen receptor beta, and GPR30 in arteries and veins of patients with atherosclerosis. Hypertension.

[28] Zhu Y, Bian Z, Lu P (2002). Abnormal vascular function and hypertension in mice deficient in estrogen receptor β. Science.

[29] Figtree GA, McDonald D, Watkins H, Channon KM (2003). Truncated estrogen receptor alpha 46-kDa isoform in human endothelial cells: relationship to acute activation of nitric oxide synthase. Circulation.

[30] Flouriot G, Griffin C, Kenealy M, Sonntag-Buck V, Gannon F (1998). Differentially expressed messenger RNA isoforms of the human estrogen receptor-alpha gene are generated by alternative splicing and promoter usage. Mol Endocrinol.

[31] Hayashi K, Maeda S, Iemitsu M (2007). Differences in the relationship between estrogen receptor alpha gene polymorphisms and arterial stiffness in older humans. Am J Hypertens.

[32] Hisamoto K, Bender JR (2005). Vascular cell signaling by membrane estrogen receptors. Steroids.

[33] Leung SW, Teoh H, Keung W, Man RY (2007). Non-genomic vascular actions of female sex hormones: physiological implications and signalling pathways. Clin Exp Pharmacol Physiol.

[34] Wynne FL, Payne JA, Cain AE, Reckelhoff JF, Khalil RA (2004). Age-related reduction in estrogen receptor-mediated mechanisms of vascular relaxation in female spontaneously hypertensive rats. Hypertension.

[35] Post WS, Goldschmidt-Clermont PJ, Wilhide CC (1999). Methylation of the estrogen receptor gene is associated with aging and atherosclerosis in the cardiovascular system. Cardiovasc Res.

[36] Bolego C, Vegeto E, Pinna C, Maggi A, Cignarella A (2006). Selective agonists of estrogen receptor isoforms: new perspectives for cardiovascular disease. Arterioscler Thromb Vasc Biol.

[37] Tsang SY, Yao X, Chan FL (2004). Estrogen and tamoxifen modulate cerebrovascular tone in ovariectomized female rats. Hypertension.

[38] Sun J, Huang YR, Harrington WR, Sheng S, Katzenellenbogen JA, Katzenellenbogen BS (2002). Antagonists selective for estrogen receptor alpha. Endocrinology.

[39] Pinna C, Bolego C, Sanvito P (2006). Raloxifene elicits combined rapid vasorelaxation and long-term anti-inflammatory actions in rat aorta. J Pharmacol Exp Ther.

[40] Mosca L, Barrett-Connor E, Wenger NK (2001). Design and methods of the Raloxifene Use for The Heart (RUTH) study. Am J Cardiol.

[41] Barrett-Connor E, Mosca L, Collins P (2006). Raloxifene Use for The Heart (RUTH) Trial Investigators Effects of raloxifene on cardiovascular events and breast cancer in postmenopausal women. N Engl J Med.

[42] Harrington WR, Sheng S, Barnett DH, Petz LN, Katzenellenbogen JA, Katzenellenbogen BS (2003). Activities of estrogen receptor alpha- and beta-selective ligands at diverse estrogen responsive gene sites mediating transactivation or transrepression. Mol Cell Endocrinol.

[43] Sun J, Meyers MJ, Fink BE, Rajendran R, Katzenellenbogen JA, Katzenellenbogen BS (1999). Novel ligands that function as selective estrogens or antiestrogens for estrogen receptor-alpha or estrogen receptor-beta. Endocrinology.

[44] Seely EW, Brosnihan KB, Jeunemaitre X (2004). Effects of conjugated oestrogen and droloxifene on the renin-angiotensin system, blood pressure and renal blood flow in postmenopausal women. Clin Endocrinol (Oxf).

[45] Xu YY, Yang C, Li SN (2006). Effects of genistein on angiotensin-converting enzyme in rats. Life Sci.

[46] Dean SA, Tan J, O'Brien ER, Leenen FH (2005). 17beta-estradiol downregulates tissue angiotensin-converting enzyme and ANG II type 1 receptor in female rats. Am J Physiol Regul Integr Comp Physiol.

[47] Dubey RK, Gillespie DG, Imthurn B, Rosselli M, Jackson EK, Keller PJ (1999). Phytoestrogens inhibit growth and MAP kinase activity in human aortic smooth muscle cells. Hypertension.

[48] Barchiesi F, Jackson EK, Gillespie DG, Zacharia LC, Fingerle J, Dubey RK (2002). Methoxyestradiols mediate estradiol-induced antimitogenesis in human aortic SMCs. Hypertension.

[49] Crews JK, Khalil RA (1999). Antagonistic effects of 17 beta-estradiol, progesterone, and testosterone on Ca^2+^ entry mechanisms of coronary vasoconstriction. Arterioscler Thromb Vasc Biol.

[50] Kauser K, Rubanyi GM (1995). Gender difference in endothelial dysfunction in the aorta of spontaneously hypertensive rats. Hypertension.

[51] Rubanyi GM, Freay AD, Kauser K (1997). Vascular estrogen receptors and endothelium-derived nitric oxide production in the mouse aorta Gender difference and effect of estrogen receptor gene disruption. J Clin Invest.

[52] Chen Z, Yuhanna IS, Galcheva-Gargova Z, Karas RH, Mendelsohn ME, Shaul PW (1999). Estrogen receptor alpha mediates the nongenomic activation of endothelial nitric oxide synthase by estrogen. J Clin Invest.

[53] Pare G, Krust A, Karas RH (2002). Estrogen receptor-alpha mediates the protective effects of estrogen against vascular injury. Circ Res.

[54] Darblade B, Pendaries C, Krust A (2002). Estradiol alters nitric oxide production in the mouse aorta through the alpha-, but not beta-, estrogen receptor. Circ Res.

[55] Nilsson BO, Ekblad E, Heine T, Gustafsson JA (2000). Increased magnitude of relaxation to oestrogen in aorta from oestrogen receptor beta knock-out mice. J Endocrinol.

[56] Widder J, Pelzer T, von Poser-Klein C (2003). Improvement of endothelial dysfunction by selective estrogen receptor-alpha stimulation in ovariectomized SHR. Hypertension.

[57] Chambliss KL, Shaul PW (2002). Estrogen modulation of endothelial nitric oxide synthase. Endocr Rev.

[58] Chambliss KL, Yuhanna IS, Anderson RG, Mendelsohn ME, Shaul PW (2002). ERbeta has nongenomic action in caveolae. Mol Endocrinol.

[59] Forte P, Kneale BJ, Milne E (1998). Evidence for a difference in nitric oxide biosynthesis between healthy women and men. Hypertension.

[60] Joy S, Siow RC, Rowlands DJ (2006). The isoflavone Equol mediates rapid vascular relaxation: Ca^2+^-independent activation of endothelial nitric-oxide synthase/Hsp90 involving ERK1/2 and Akt phosphorylation in human endothelial cells. J Biol Chem.

[61] Haynes MP, Sinha D, Russell KS (2000). Membrane estrogen receptor engagement activates endothelial nitric oxide synthase *via* the PI3-kinase-Akt pathway in human endothelial cells. Circ Res.

[62] Hernández I, Delgado JL, Díaz J (2000). 17beta-estradiol prevents oxidative stress and decreases blood pressure in ovariectomized rats. Am J Physiol Regul Integr Comp Physiol.

[63] Barber DA, Miller VM (1997). Gender differences in endothelium-dependent relaxations do not involve NO in porcine coronary arteries. Am J Physiol.

[64] Egan KM, Lawson JA, Fries S (2004). COX-2-derived prostacyclin confers atheroprotection on female mice. Science.

[65] Bilsel AS, Moini H, Tetik E, Aksungar F, Kaynak B, Ozer A (2000). 17Beta-estradiol modulates endothelin-1 expression and release in human endothelial cells. Cardiovasc Res.

[66] Dubey RK, Jackson EK, Keller PJ, Imthurn B, Rosselli M (2001). Estradiol metabolites inhibit endothelin synthesis by an estrogen receptor-independent mechanism. Hypertension.

[67] David FL, Carvalho MH, Cobra AL (2001). Ovarian hormones modulate endothelin-1 vascular reactivity and mRNA expression in DOCA-salt hypertensive rats. Hypertension.

[68] Collins P, Rosano GM, Sarrel PM (1995). 17 beta-Estradiol attenuates acetylcholine-induced coronary arterial constriction in women but not men with coronary heart disease. Circulation.

[69] Nickenig G, Strehlow K, Wassmann S (2000). Differential effects of estrogen and progesterone on AT(1) receptor gene expression in vascular smooth muscle cells. Circulation.

[70] Salamanca DA, Khalil RA (2005). Protein kinase C isoforms as specific targets for modulation of vascular smooth muscle function in. Hypertension Biochem Pharmacol.

[71] Murphy JG, Khalil RA (2000). Gender-specific reduction in contractility and [Ca^2+^]_i_ in vascular smooth muscle cells of female rat. Am J Physiol Cell Physiol.

[72] Murphy JG, Khalil RA (1999). Decreased [Ca^2+^]_i_ during inhibition of coronary smooth muscle contraction by 17beta-estradiol, progesterone, and testosterone. J Pharmacol Exp Ther.

[73] Han G, Yu X, Lu L (2006). Estrogen receptor alpha mediates acute potassium channel stimulation in human coronary artery smooth muscle cells. J Pharmacol Exp Ther.

[74] Prakash YS, Togaibayeva AA, Kannan MS, Miller VM, Fitzpatrick LA, Sieck GC (1999). Estrogen increases Ca^2+^ efflux from female porcine coronary arterial smooth muscle. Am J Physiol.

[75] Kanashiro CA, Khalil RA (2001). Gender-related distinctions in protein kinase C activity in rat vascular smooth muscle. Am J Physiol Cell Physiol.

[76] Somlyo AP, Somlyo AV (2000). Signal transduction by G-proteins, rho-kinase and protein phosphatase to smooth muscle and non-muscle myosin II. J Physiol.

[77] Lee DL, Webb RC, Jin L (2004). Hypertension and RhoA/Rho-kinase signaling in the vasculature: highlights from the recent literature. Hypertension.

[78] Noma K, Oyama N, Liao JK (2006). Physiological role of ROCKs in the cardiovascular system. Am J Physiol Cell Physiol.

[79] Shimokawa H, Takeshita A (2005). Rho-kinase is an important therapeutic target in cardiovascular medicine. Arterioscler Thromb Vasc Biol.

[80] Chrissobolis S, Budzyn K, Marley PD, Sobey CG (2004). Evidence that estrogen suppresses rho-kinase function in the cerebral circulation *in vivo*. Stroke.

[81] Simoncini T, Scorticati C, Mannella P (2006). Estrogen receptor alpha interacts with Galpha13 to drive actin remodeling and endothelial cell migration *via* the RhoA/Rho kinase/moesin pathway. Mol Endocrinol.

[82] Groten T, Pierce AA, Huen AC, Schnaper HW (2005). 17 beta-estradiol transiently disrupts adherens junctions in endothelial cells. FASEB J.

[83] Felty Q, Roy D (2005). Estrogen, mitochondria, and growth of cancer and non-cancer cells. J Carcinog.

[84] Cid MC, Esparza J, Schnaper HW (1999). Estradiol enhances endothelial cell interactions with extracellular matrix proteins *via* an increase in integrin expression and function. Angiogenesis.

[85] Wingrove CS, Garr E, Godsland IF, Stevenson JC (1998). 17beta-oestradiol enhances release of matrix metalloproteinase-2 from human vascular smooth muscle cells. Biochim Biophys Acta.

[86] Sumino H, Ichikawa S, Kasama S (2005). Effect of transdermal hormone replacement therapy on carotid artery wall thickness and levels of vascular inflammatory markers in postmenopausal women. Hypertens Res.

[87] Gao H, Liang M, Bergdahl A (2006). Estrogen attenuates vascular expression of inflammation associated genes and adhesion of monocytes to endothelial cells. Inflamm Res.

[88] Geraldes P, Gagnon S, Hadjadj S (2006). Estradiol blocks the induction of CD40 and CD40L expression on endothelial cells and prevents neutrophil adhesion: an ERalpha-mediated pathway. Cardiovasc Res.

[89] Xing D, Feng W, Miller AP (2007). Estrogen modulates TNF-alpha-induced inflammatory responses in rat aortic smooth muscle cells through estrogen receptor-beta activation. Am J Physiol Heart Circ Physiol.

[90] Arenas IA, Armstrong SJ, Xu Y, Davidge ST (2006). Tumor necrosis factor-alpha and vascular angiotensin II in estrogen-deficient rats. Hypertension.

[91] Brandes RP, Fleming I, Busse R (2005). Endothelial aging. Cardiovasc Res.

[92] Duckles SP, Krause DN, Stirone C, Procaccio V (2006). Estrogen and mitochondria: a new paradigm for vascular protection?. Mol Interv.

[93] Yeboah J, Reboussin DM, Waters D, Kowalchuk G, Herrington DM (2000). Effects of estrogen replacement with and without medroxyprogesterone acetate on brachial flow-mediated vasodilator responses in postmenopausal women with coronary artery disease. Am Heart J.

[94] Mikkola TS, Clarkson TB (2002). Estrogen replacement therapy, atherosclerosis, and vascular function. Cardiovasc Res.

[95] López-Jaramillo P, Díaz LA, Pardo A, Parra G, Jaimes H, Chaudhuri G (2004). Estrogen therapy increases plasma concentrations of nitric oxide metabolites in postmenopausal women but increases flow-mediated vasodilation only in younger women. Fertil Steril.

[96] Sherwood A, Bower JK, McFetridge-Durdle J, Blumenthal JA, Newby LK, Hinderliter AL (2007). Age moderates the short-term effects of transdermal 17beta-estradiol on endothelium-dependent vascular function in postmenopausal women. Arterioscler Thromb Vasc Biol.

[97] Naftolin F, Taylor HS, Karas R (2004). Women's Health Initiative The Women's Health Initiative could not have detected cardioprotective effects of starting hormone therapy during the menopausal transition. Fertil Steril.

[98] Hsia J, Langer RD, Manson JE (2006). Women's Health Initiative Investigators. Conjugated equine estrogens and coronary heart disease: the Women's Health Initiative. Arch Intern Med.

[99] Harman SM, Brinton EA, Cedars M (2005). KEEPS: The Kronos Early Estrogen Prevention Study. Climacteric.

[100] Kelemen M, Vaidya D, Waters DD (2005). Hormone therapy and antioxidant vitamins do not improve endothelial vasodilator function in postmenopausal women with established coronary artery disease: a substudy of the Women's Angiographic Vitamin and Estrogen (WAVE) trial. Atherosclerosis.

[101] Chataigneau T, Zerr M, Chataigneau M (2004). Chronic treatment with progesterone but not medroxyprogesterone acetate re-stores the endothelial control of vascular tone in the mesenteric artery of ovariectomized rats. Menopause.

[102] Minshall RD, Pavcnik D, Browne DL, Hermsmeyer K (2002). Nongenomic vasodilator action of progesterone on primate coronary arteries. J Appl Physiol.

[103] Wassmann K, Wassmann S, Nickenig G (2005). Progesterone antagonizes the vasoprotective effect of estrogen on antioxidant enzyme expression and function. Circ Res.

[104] Sunday L, Tran MM, Krause DN, Duckles SP (2006). Estrogen and progestagens differentially modulate vascular proinflammatory factors. Am J Physiol Endocrinol Metab.

[105] Faludi AA, Aldrighi JM, Bertolami MC (2004). Progesterone abolishes estrogen and/or atorvastatin endothelium dependent vasodilatory effects. Atherosclerosis.

[106] Adams MR, Register TC, Golden DL, Wagner JD, Williams JK (1997). Medroxyprogesterone acetate antagonizes inhibitory effects of conjugated equine estrogens on coronary artery atherosclerosis. Arterioscler Thromb Vasc Biol.

[107] Wynne FL, Khalil RA (2003). Testosterone and coronary vascular tone: implications in coronary artery disease. J Endocrinol Invest.

[108] Montalcini T, Gorgone G, Gazzaruso C, Sesti G, Perticone F, Pujia A (2007). Endogenous testosterone and endothelial function in postmenopausal women. Coron Artery Dis.

[109] Ding AQ, Stallone JN (2001). Testosterone-induced relaxation of rat aorta is androgen structure specific and involves K+ channel activation. J Appl Physiol.

[110] Honda H, Unemoto T, Kogo H (1999). Different mechanisms for testosterone-induced relaxation of aorta between normotensive and spontaneously hypertensive rats. Hypertension.

[111] Norata GD, Tibolla G, Seccomandi PM, Poletti A, Catapano AL (2006). Dihydrotestosterone decreases tumor necrosis factor-alpha and lipopolysaccharide-induced inflammatory response in human endothelial cells. J Clin Endocrinol Metab.

[112] Song D, Arikawa E, Galipeau D, Battell M, McNeill JH (2004). Androgens are necessary for the development of fructose-induced. Hypertension Hypertension.

[113] Zhai P, Eurell TE, Cooke PS, Lubahn DB, Gross DR (2000). Myocardial ischemia-reperfusion injury in estrogen receptor-alpha knockout and wild-type mice. Am J Physiol Heart Circ Physiol.

[114] Wang M, Crisostomo P, Wairiuko GM, Meldrum DR (2006). Estrogen receptor-alpha mediates acute myocardial protection in females. Am J Physiol Heart Circ Physiol.

[115] Hinojosa-Laborde C, Craig T, Zheng W, Ji H, Haywood JR, Sandberg K (2004). Ovariectomy augments hypertension in aging female Dahl salt-sensitive rats. Hypertension.

[116] Crews JK, Murphy JG, Khalil RA (1999). Gender differences in Ca^2+^ entry mechanisms of vasoconstriction in Wistar-Kyoto and spontaneously hypertensive rats. Hypertension.

